# Satellite observation of atmospheric CO_2_ and water storage change over Iran

**DOI:** 10.1038/s41598-023-28961-x

**Published:** 2023-02-21

**Authors:** Samaneh Safaeian, Samereh Falahatkar, Mohammad J. Tourian

**Affiliations:** 1 Department of Environmental Sciences, Natural Resources Faculty, Tarbiat Modares University, Noor, Mazandaran Iran; 2 Institute of Geodesy, University of Stuttgart, Stuttgart, Germany

**Keywords:** Environmental impact, Climate change

## Abstract

Like many other Middle East countries, Iran has been suffering from severe water shortages over the last two decades, as evidenced by significant decline in surface water and groundwater levels. The observed changes in water storage can be attributed to the mutually reinforcing effects of human activities, climatic variability, and of course the climate change. The objective of this study is to analyze the dependency of atmospheric CO_2_ increase on the water shortage of Iran, for which we investigate the spatial relationship between water storage change and CO_2_ concentration using large scale satellite data. We conduct our analysis using water storage change data from GRACE satellite and atmospheric CO_2_ concentration from GOSAT and SCIAMACHY satellites during 2002–2015. To analyze the long-term behavior of time series we benefit from Mann-Kendal test and for the investigation of the relationship between atmospheric CO_2_ concentration and total water storage we use Canonical Correlation Analysis (CCA) and Regression model. Our Results show that the water storage change anomaly and CO_2_ concentration are negatively correlated especially in northern, western, southwest (Khuzestan province), and also southeast (Kerman, Hormozgan, Sistan, and Baluchestan provinces) of Iran. CCA results reveal that in the most of northern regions, the decrease in water storage is significantly influenced by the increase of CO_2_ concentration. The results further show that precipitation in the highland and peaks does not seem to be influenced by the long and short-term variation in CO_2_ concentration. Besides, our results show that the CO_2_ concentration is slightly correlated with a weak positive trend in evapotranspiration over agricultural areas. Thus, the indirect effect of CO_2_ on increasing evapotranspiration is observed spatially in the whole of Iran. The results of the regression model between total water storage change and carbon dioxide (R^2^ = 0.91)/water discharge/water consumption show that carbon dioxide has the highest effect on total water storage change at large scale. The results of this study will contribute to both water resource management and mitigation plans to achieve the goal of CO_2_ emission reduction.

## Introduction

The Earth’s average temperature (15 degrees Celsius) increases since the Industrial Revolution. NASA has reported an average increase of global surface temperatures by 0.9 °C from 1880 to 2015 (NASA’S Goddard Institute). Carbon dioxide (CO_2_) is identified as the most important greenhouse gas in the atmosphere^1^ and its concentration in the troposphere has been increased from about 277 ppm in 1750 to 408.12 ppm in 2019^2,3^). The reduction of carbon dioxide (CO_2_) emissions by 20%, is one of the so-called 20/20/20 targets, within the Paris Agreement with the long-term goal of keeping the increase in global average temperature well below 2 °C above pre-industrial levels. Water and carbon cycles are recognized as life components on Earth and play an important role in climate change and water/food security^4^. The exchange of CO_2_ between different ecosystems and the atmosphere has a significant role in the earth’s temperature and changes in climatic characteristics effect on the hydrological cycle^5^. As atmospheric CO_2_ concentrations increase, the climate and hydrologic cycle are changing, and their consequences on temperature, cloud cover, evapotranspiration, runoff formation, and precipitation intensity and distribution are observed in natural ecosystems^4,5^.

The climate of the Middle East is arid and hot. Water scarcity has been identified as the biggest challenge particularly since the onset of drought in 2007. Based on the World Bank statistics in 2007, about 50% of the countries in the Middle East are consuming more water on average than they receive in precipitation, and 85% of total surface and groundwater was consumed in the agricultural sector. The desertification phenomenon is occurring over the Middle East, especially in Syria, Iran, Jordan, and Iraq. Water resources of Iran are under high stress due to population growth, land-use change such as urbanization, agricultural expansion, and their related consequences in recent decades^6^. The indirect effect of the increasing population (~ 83 million in 2019) on water resources was an increase in demand for cropland and development of irrigation lands while its direct effect resulted in an increased necessity for fresh water in the urban and rural areas^7^. Sarraf et al.^8^ stated that the total water storage over Iran have been decreased more than 65% since 1960, and a reduction of 16% will expect to 2025. Over-exploitation of water resources (surface and groundwater) and the high rate of irrigation in some provinces are the main causes of challenge for the future preservation of groundwater basins in most parts of Iran^9^, Mohammadi Ghaleni & Ebrahimi^10^). It is worth mentioning that, approximately 50% of the water volume of irrigation is supported from surface sources such as lakes and rivers and the other 50% is provided from groundwater^7^. Over the past three decades, an over-exploitation of groundwater resources has caused a drop in water level in the majority of basins in Iran^9^. Also, some activities such as industrial and agricultural activities have caused a severe decrease in groundwater quality in most parts of Iran^11^. In the interim, evaporation phenomena could rise the salinity of cropland soil and surface waters, typically in rivers and natural lakes.

The studying bilateral relationship between atmospheric CO_2_ amplitude and water cycle is an interesting topic in environmental sciences and climate change. In this research, for the first time, we try to use remote sensing technology advances for investigating the relationship between atmospheric CO_2_ and inland water storage on a regional scale. Among them, space-borne geodetic sensors, planed for various purposes, have established themselves as a useful instrument for oceanographic, cryospheric, and also hydrological applications. For example, radar satellite altimetry, originally designed for applications in oceanography and geodesy, has indicated its potential as virtual lake and river gauges^12^, Papa et al.^13^; Refs.^14–16^. The launch of Gravity Recovery and Climate Experiment (GRACE) in 2002^17^ allowed for the recovery of the time variable gravity field at catchment scales using low-low satellite to satellite tracking (llSST). GRACE data has provided a direct estimation of the monthly gravity field variations through calculation of the relative motion of the center-of-mass of the twin satellites with a highly accurate inter-satellite K-band microwave link. GRACE is a special mission among all other space-borne sensors as it allows us to investigate continental water storage on short time scales^18^. Many types of research were devoted to use GRACE data for analysis of equivalent water height (EWH) change^19,17^, Forotan et al.^20^, Ref.^21–23^. The presence of such a satellite mission motivated this study to understand the relationship between the total water storage changes, as seen by GRACE, and the CO_2_ concentration as also measured from space.

Greenhouse gas monitoring using remote sensing technologies began in 2002 by SCIAMACHY onboard ENVISAT and continues by GOSAT, OCO-2, and Tansat^24^, Schneising et al.^25^, Ref.^26,27^. For example, Shim et al.^28^ used GOSAT satellite data in their study area to investigate the greenhouse gas emissions in East Asia between April 2009 and 2011. They have shown that the highest concentration of carbon dioxide in April is that plants have not yet started to grow. The lowest amount is in July and August when photosynthetic intensity reaches its highest level. Jing et al.^29^ prepared a mapping of global carbon dioxide distribution in September 2012 using the conventional Kriging interpolation method and combined use of GOSAT and SCIAMACHY satellite data. Sun et al.^30^ used carbon dioxide spatial data from the GOSAT satellites (July 2009 to April 2014) and ENVISAT as well as monthly mean atmospheric carbon dioxide emissions from Loa Manua and MEI. The objectives of their study were to determine the relationship between atmospheric CO_2_ concentration and ENSO and also to investigate variations in the spatial distribution of CO_2_ concentration in South America during different phases of ENSO. They showed that the average monthly carbon dioxide growth rate is positively correlated with MEI. Mousavi et al.^31^ used near-infrared thermal sensors for Fourier Carbon Spectroscopy Observations (TANSO-FTS) from GOSAT level-2 data and meteorological parameters to estimate CO_2_ variations from 2009 to 2015. They expressed that CO_2_ variations are highly dependent on the month, with the highest CO_2_ concentrations in April and May and the lowest concentrations in August and September. The correlation between XCO_2_ and the monthly mean air temperature was also shown to be negative, indicating that the decrease in XCO_2_ with temperature increases depends on the photosynthetic process by vegetation in the hot season. Falahatkar et al.^32^ studied the relationship between XCO_2_, normalized difference vegetation index, and some meteorological variables such as air temperature, humidity, and rainfall on a national scale. Their findings showed that the highest and lowest values of carbon dioxide concentration were distributed in the southeast, north and northwestern parts of Iran, respectively. In a similar study, Siabi et al.^33^ assessed the spatial distribution of atmospheric CO_2_ from April to September in the whole of Iran. They used OCO-2 data and different environmental variables for finding the relationship between atmospheric CO_2_ and environmental parameters.

Velicogna et al.^34^ used GRACE data to investigate the relationship between total water storage variation and vegetation growth. Their results showed that the availability of total water storage plays a key role in adjusting the response of vegetation to temperature fluctuations. Similar to the idea of our study, Humphrey et al.^35^ used EWH changes extracted from GRACE to study inland water effects on carbon cycle dynamics at large scales. They showed that CO_2_ change rate is significantly dependent on obtained changes in inland water storage, where drier years have been observed with a faster increasing trend of CO_2_. Further, they demonstrated that the universal relationship is independent of known air temperature effects and is underestimated in current carbon cycle models. Gentine et al.^5^ explored the key role of inland water availability in the carbon cycle and highlighted the effective role of satellites in studying the coupling between the atmospheric carbon dioxide and water cycles.

Although the change in total water storage has been studied using both remote sensing data and in situ data with climatic characteristics and economic parameters, to the best of our knowledge, there is no study that examines the bilateral relationship between CO_2_ and change in water storage in Iran. We recall that terrestrial carbon sinks reduce with increasing drought events and water shortage, as has been the case in Iran since 2008. Therefore, we aim to investigate the link between total water storage and CO_2_ change in the time. To this end, for the first time, we perform a careful analysis of dependency between equivalent water height anomaly by GRACE data and atmospheric CO_2_ concentration during last 15 years. Our results are important and beneficial for future studies on hydrology, ecology, and climate change in Iran and will be essential for both water resources management and CO_2_ mitigation plans. Such an analysis includes the following steps, which also characterize the objectives of this study:

1. The time series analysis of total water storage and CO_2_ concentration change during 15 years,
2. Quantification the relationship between total water storage and atmospheric CO_2_ in national scale,
3. Finding the relationship between total water change and atmospheric CO_2_/ water discharge/water consumption over Iran.

## Study area

**Figure 1:**
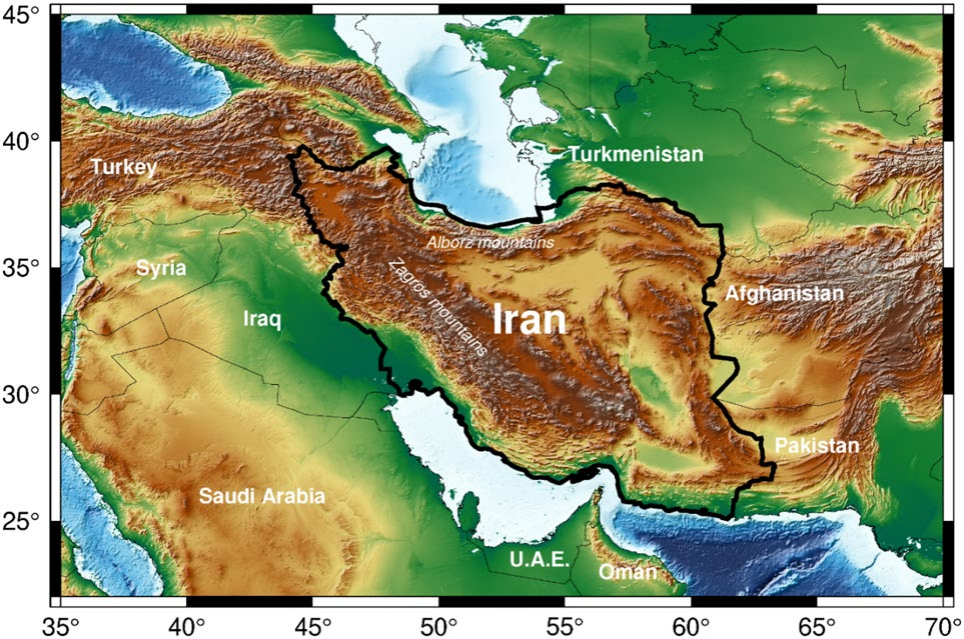
The location of Iran in the Middle East and its neighboring countries. It is generated using the Generic Mapping Tools (GMT).

Iran is located between 25° to 40°N latitude and 44° to 63°E longitude and has an area of 1,648,000 km^2^ (Fig. 1). Iran is known as an arid and semi-arid country. The elevation range is − 30 m to more than 5500 m, which has a significant influence on variety of climates. However, Iran has a large diversity of climatic regions with precipitation range (average of 120 mm yr^−1^ in the central and eastern provinces and averages of 2000 mm/yr in the northern regions of Iran) and temperature variation (range of – 20 °C in the northwest to 50 °C over southern Iran). There are two famous mountains in Iran. Alborz and Zagrous mountains are located in northern and western Iran. More than 50% of the area between Alborz and Zagrous is covered by salty swamps of Dasht-e-Kavir and Kavir-e-Lut. The entire Iran is constituted by six main catchments. The Caspian Sea, Urmia Lake, the Persian Gulf and the Gulf of Oman, the central plateau, the Kara-Kum, and the Lake Hamoon are located in northern, northwestern, southern, central, northeastern, and eastern Iran.

## Data

### GRACE equivalent water height anomaly

The Gravity Recovery and Climate Experiment) GRACE) is a satellite mission to produce the global gravity field map in a spatial resolution of about 400 km every 30 days^36,37^. GRACE includes two identical satellites in 500 km altitude and 89.5° inclination, separated from each other by about 220 km along-track. GRACE presents a direct estimation of the monthly gravity field variations by observing the relative motion of the center-of-mass of the two satellites, which is measured with the highly accurate inter-satellite K-band microwave link. This mensuration is obtained by gravity variability over time on the land, which represents change in water mass after accounting for aliased signals and under certain considerations of temporal and spatial resolution^38^. In this study, we used data from level-2 of the GRACE satellites (Mascon) in the period from 2003 to 2015. The JPL RL06M Mascon solution provides for monthly gravity field variations in terms of equal-area 3 by 3 degree spherical cap Mascons. The data are available in the form of Equivalent Water Height (EWH) anomaly in mm for 0.5 degree cells and they were downloaded from https://grace.jpl.nasa.gov/data/get-data/jpl_global_mascons (Table 1).

### XCO_2_ Obs4MIPs product

We use the mean of XCO_2_ (Mole fraction of tropospheric CO_2_) data sets that retrieved from SCIAMACHY and TANSO-FTS sensors that onboard ENVISAT and GOSAT satellites, respectively^39,40^. XCO_2_ has no dimension (unit: mol/mol) explained as number of CO_2_ molecule divided by number of dry air molecule in an air vertical column. The Obs4MIPs product has been produced using Level-2 XCO_2_ products as input. Note that a resolution of 5° × 5° has been selected to ensure better noise control. In this study, GOSAT and SCIAMACHY were combined with Obs4MIPs algorithm and 2003 to 2015. Data were downloaded from Institute of Environmental Physics, University of Bremen (Table 1).

### Precipitation and evaporation

We use the monthly data sets of the ERA5 dataset for both precipitation and evapotranspiration (https://www.ecmwf.int/en/forecasts/datasets/reanalysis-datasets/era5). ERA5 dataset include hourly different climate parameters in atmosphere, hydrosphere and land, which is available from 1950 to present (Copernicus Climate Change Service (C3S)^41^) (Table 1).

Apart from above mentioned datasets, for the plausibility assessment of our results, we relied on data like statistics of piezometric wells, water discharge (springs, aqueducts, deep wells, semi-deep wells) and water consumption in three sectors (agriculture, industry and domestic) which were obtained from the site of Iran Water Resources Studies.

**Table 1:** Lists all the datasets used in this study, which are explained in the following sections.

Data	Product	Sensor/method	Resolution	Level	Source
Equivalent water height	Mascon solution	GRACE	0.5 degree	2	https://grace.jpl.nasa.gov/data/get-data/jpl_global_mascons
CO_2_ concentration	XCO_2_ Obs4MIPs product	GOSAT and SCIAMACHY	5 degree	2	Institute of Environmental Physics, University of Bremen
Precipitation	ERA5	Reanalysis	0.25 degree	–	https://www.ecmwf.int/en/forecasts/datasets/reanalysis-datasets/era5
Evapotranspiration	ERA5	Reanalysis	31 km	–	https://www.ecmwf.int/en/forecasts/datasets/reanalysis-datasets/era5

## Methodology

Our analysis of relationship between CO_2_ and water storage relies on, first of all, performing a time series analysis to understand the statistical behavior of these time series and their long-term trend. Moreover, to capture the related patterns of two datasets, we used a Canonical Correlation Analysis (CCA).

### Time series analysis

For the time series analysis, apart from the graphical inspection on time series and correlation analysis, we perform the Mann-Kendal trend test. Kendall and Ord (1990) described trend as a long-term movement. The monotonic trend (Mann–Kendall) provides a non-linear trend indicator that estimates the degree to which a trend is continuously increasing or decreasing. Mann–Kendall values have a range from negative one to positive one. A value of + 1 expresses a trend that consistently increases and does not have any decreases in study period. A value of − 1 shows consistently decrease during the study period. The zero value represents no consistent trend. The Theil-Sen slope is estimated based on median trend. All pairwise combinations of values are assessed for each pixel during study time and a tally is made of the number that are increasing or are decreasing with time. The Mann–Kendall significance produces two maps; a significance map represented as Z scores and a second image that indicates the probability (P value) that the obtained trend could have occurred by chance. Moreover, P value represent the significance of a Mann–Kendall trend over time^42^.

### Canonical correlation analysis

Canonical correlation analysis (CCA) is a method for extracting joint modes between two time series variables^43^. This method extracts the bilateral information of two time series datasets through performing unique value decomposition on the covariance matrix between two data sets. This Method can detect the basis vectors for two different time series datasets without considering the units, as it relies on correlation between datasets that is invariant to the magnitude. Unlike PCA, CCA considers maximum value of covariance between variables not maximum value of variance^44^, Zhang et al.^45^). The bilateral correlated information are obtained in different canonical modes of both fields. CCA distinguishes relationships between two field series to identify related patterns between the series.

CCA as employed here can be referred to as a double-barreled PCA^42^. Two variables X (t_1_ × n_1_) and Y (t_2_ × n_2_) as atmospheric CO_2_ concentration and EWH anomaly, respectively, at n_1_ and n_2_ grid points in spatial characteristics, taken at time 1 and time 2. It should be pointed out that one of the dimensions of two variables (X and Y) must have the same size. Therefore, time was considered as same dimension of X and Y variables. We estimate the covariance matrix X^T^Y (n_1_ × n_2_) between X and Y variables to which unique value decomposition is now applied

1 $${\text{C}}_{{{\text{XY}}}} = {\text{X}}^{{\text{T}}} {\text{Y }} = {\text{ U}}_{{\text{C}}} \cdot \, \Sigma_{{\text{C}}} \cdot {\text{ V}}_{{\text{C}}}^{{\text{T}}} .$$

In Eq. (1), U_C_, V_C_ are canonical modes, and Σ_C_ (n_1_ × n_2_) represents the singular values of the covariance between both time series. U_C_, V_C_ with n_1_, n_2_ dimension indicate spatial information. With the canonical modes for each time series dataset, the corresponding associated components are then calculated by prediction the data set on them (Eqs. (2) and (3)):

2 $${\text{V}}_{{\text{X}}} = {\text{X}} \cdot {\text{U}}_{{\text{C}}} ,$$

3 $${\text{V}}_{{\text{Y}}} = {\text{Y}} \cdot {\text{V}}_{{\text{C}}} ,$$

where V_X_ (t × r) and V_Y_ (t × r) are the associated temporal characteristics of the time series X and Y with dimension time (t), respectively. Also, C_XY_ is the calculated time independently, thus U_C_, V_C_ indicate the spatial pattern, and V_X_, V_Y_ consequently refer to temporal correlated information. A subgroup of the canonical modes of both time series is considered based on the highest variance.

### Regression modeling

One of the main goals of statistical studies is to find the relationship between two or more variables. Regression methods refer to a method of statistical modeling that analyzes such relationships (Adab^46^). In this research, we used stepwise regression modeling for finding the relationship between total water/groundwater change and atmospheric CO_2_/water discharge/water consumption. For more details about stepwise regression modeling read Farajzadeh^47^.

## Results and discussions

### Time series analysis

**Figure 2:**
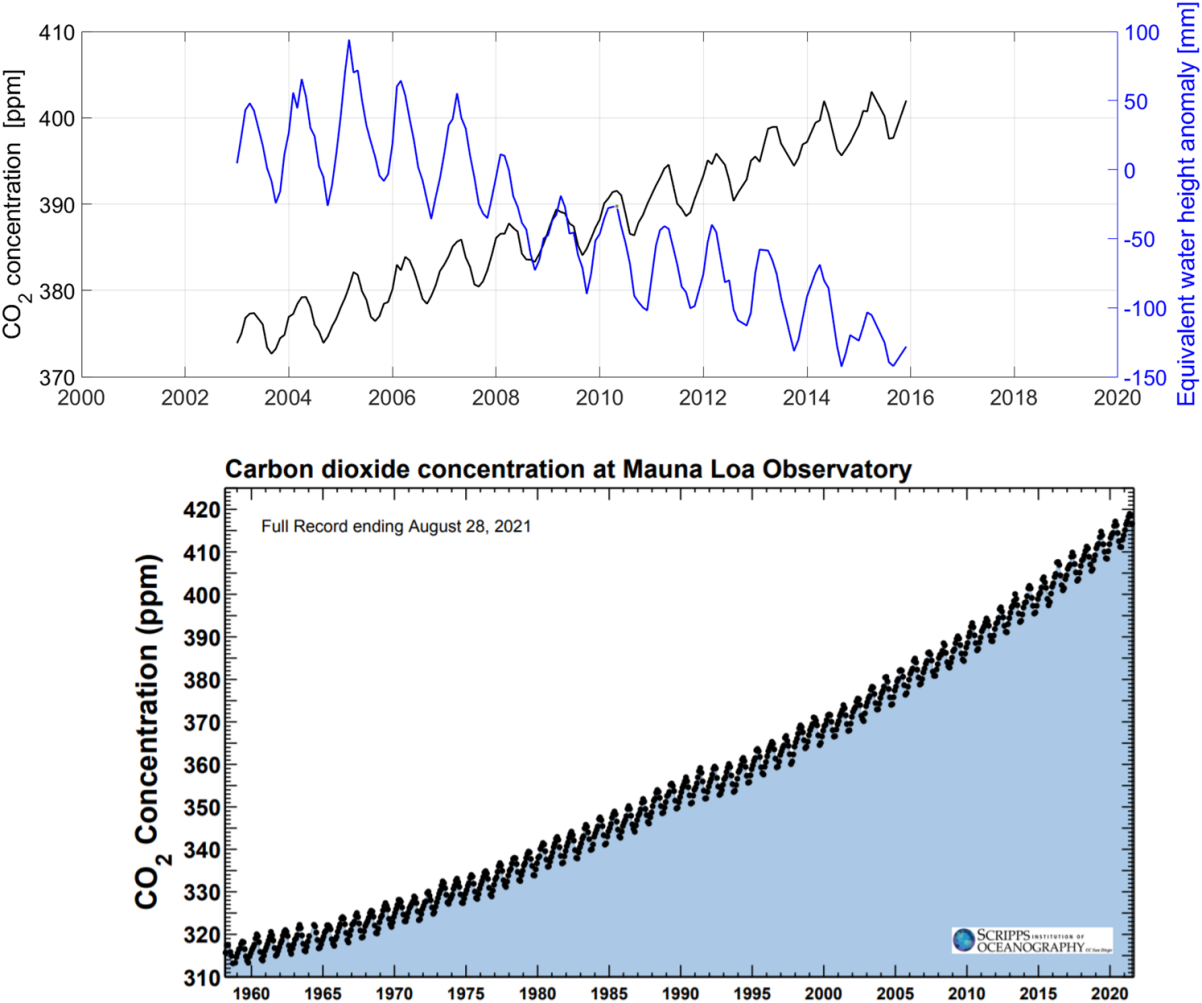
Time series of EWH anomaly and CO_2_ concentration from 2003 to 2015 over Iran (top) and full record of CO_2_ concentration (Scripps Institution of Oceanography at UC San Diego) (bottom).

**Figure 3:**
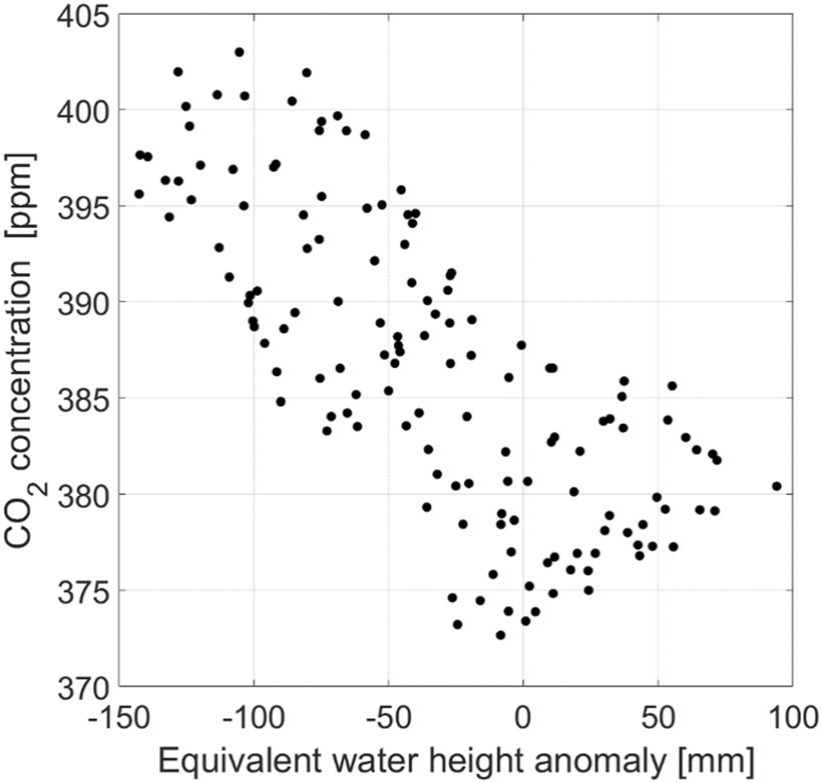
Scatter plot of CO_2_ concentration (Axis Y) versus EWH anomaly (Axis X) for 2003 to 2015.

Figure 2 shows the time series of EWH anomaly together with the time series of CO_2_ concentration over the entire Iran. The upward trend is accompanied by seasonal fluctuations in atmospheric carbon dioxide concentrations is represented from 2003 to 2015. The highest and lowest carbon dioxide concentrations are observed in spring and summer, respectively. This result is consistent with Keeling curve^3^. During this period time, sinusoidal and decreasing oscillations is exhibited the changes in the time series of EWH anomaly as well. The time series show a correlation coefficient of – 0.74 (R^2^ = 0.55), representing that the increasing carbon dioxide concentration in atmosphere of Iran accompanied with a decrease in EWH anomaly. The negative correlation is predominantly due to the negative sign in the trend of these time series. However, in terms of seasonality both time series show a positive correlation. The de-trended time series show a correlation coefficient of + 0.7 (R^2^ = 0.5), indicating a strong coupling between CO_2_ and EWH anomaly (Fig. 3).

The time series of EWH anomaly shows a negative trend of 13.6 mm/y between 2003 and 2015. On the other hand, the time series of CO_2_ concentration shows a positive trend of 2.1 ppm/yr for the same time period.

**Figure 4:**
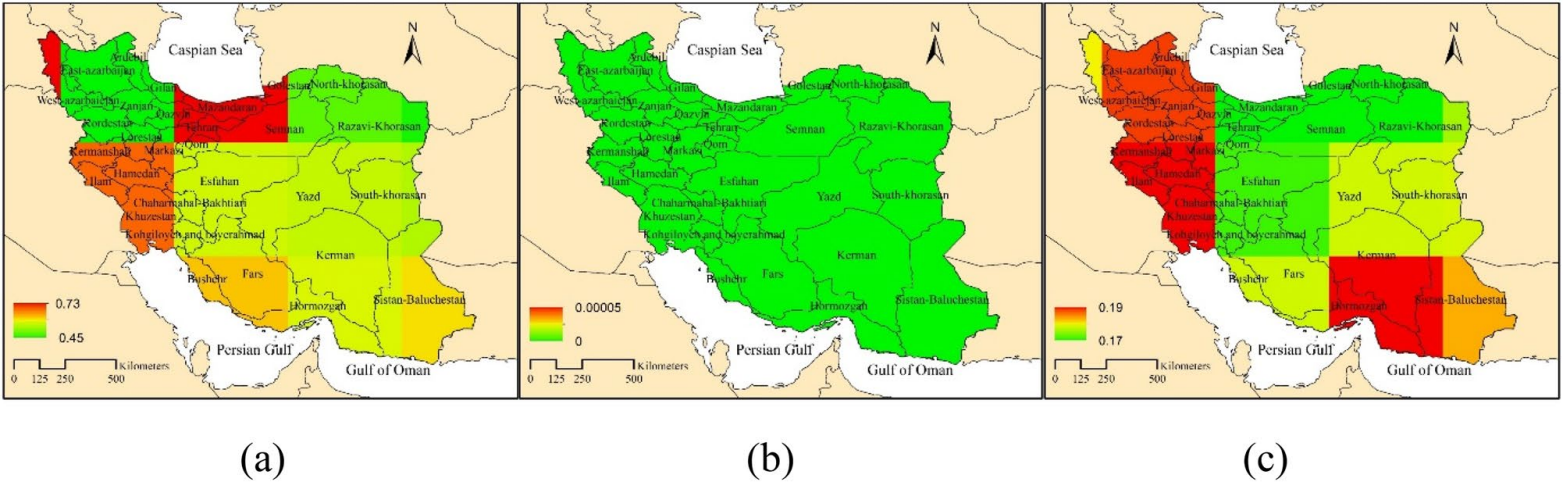
Time series trend analysis of carbon dioxide concentration using Mann–Kendall test (**a**), P value (**b**) and Theil-Sen slope (**c**).

The results of Mann Kendall test in Fig. 4a, clearly indicate an increasing trend of carbon dioxide in all parts of Iran. The highest value of monotonic Mann–Kendall was observed in northern, western, southwest (Khuzestan province) and then southeastern Iran (Kerman, Hormozgan, Sistan, and Baluchestan provinces). The lowest monotonic Mann–Kendall value was observed in the northwest of the country. According to the p-value (Fig. 4b) one can state that a significant increasing trend was observed over Iran from 2003 to 2015. The range of Theil-Sen slope was estimated very low for the variation of CO_2_ concentration in Iran (about 0.02). In the northwest and west of Iran, as well as in the south and southeast of the country, the change rate of carbon dioxide concentration is higher than in other areas (Fig. 4c). As also stated by Mousavi et al.^31^, the variation of carbon dioxide concentration is highly related to the season (Fig. 2). In fact, the highest concentration of CO_2_ in April and May and the lowest concentration in August and September^48^ observed the highest value of XCO_2_ in cold season and lowest value in the hot season based on OCO-2 data in Brezil. Mousavi et al.^49^ attributed the high carbon dioxide concentration in the eastern and southeastern parts of Iran to the high temperature and lack of dense vegetation in this area. Moreover, anthropogenic activities like deforestation, land clearing, agricultural activities, and biomass burning accelerate the process of greenhouse gas emissions^50^. Some researchers have found that land degradation caused by unbearable agricultural practices is the main reason for increasing greenhouse gas emissions.

**Figure 5:**
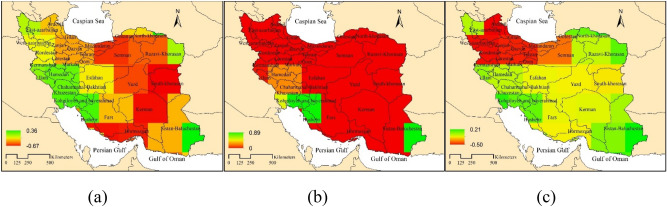
Time Series Trend of EWH anomaly Using the Man-Kendall test (**a**), P value (**b**), Theil-Sen slope (**c**).

A similar analysis is performed on EWH anomaly over Iran. In general, only part of western and southwestern Iran has been shown a less decreasing trend than other parts of Iran. The lowest decreasing trend was observed in Kohkiluyeh and Boyer-Ahmad province and then in Khuzestan and in part of Bushehr province. It is valuable to note that the highest decreasing trend was observed in Semnan province, South Khorasan province and then in Khorasan Razavi, Yazd, and Kerman provinces (Fig. 5a). The p-value for this parameter indicated that there is a significant decreasing trend (p ≤ 0.05) in all parts of Iran, except for a small part in Sistan and Baluchestan province and a small part in Kohkiloyeh and Boyer Ahmad, Bushehr, and Khuzestan provinces (Fig. 5b). According to the rate of monthly changes of EWH anomaly results, the high change rate is observed in the region including Khuzestan province and east of Sistan and Baluchestan province (Fig. 5c). Abou Zaki et al.^51^ showed the level of groundwater was decreased about 7.6 mm annual from 2002 to 2011 that equal to the total water volume 2.6 km^3^ in Bakhtegan basin. Moshir Panahi et al.^52^ sowed Iran warmed and precipitation decreased in most region and total water storage depleted from 1986 to 2016. The reason for such drastic water loss could be drought, population growth and economic growth which led to increased demand for limited water resources^20,53^, Shamsi and Ghorbani^54^, Moshir Panahi et al.^52^, and Abou Zaki et al.^51^.

### Canonical correlation analysis

The canonical correlation analysis (CCA) was applied to find out the relationship between two multidimensional variables. We have performed the analysis for the following pairs:

- CO_2_ concentration and EWH anomaly.
- CO_2_ concentration and precipitation.
- CO_2_ concentration and evapotranspiration.

In order to investigate the patterns between CO_2_ and hydrological variables, in the following, we analyze its canonical correlation with precipitation as the input and also evapotranspiration as it links Earth’s water, energy and carbon cycle.

#### CCA between CO_2_ and EWH anomaly

**Figure 6:**
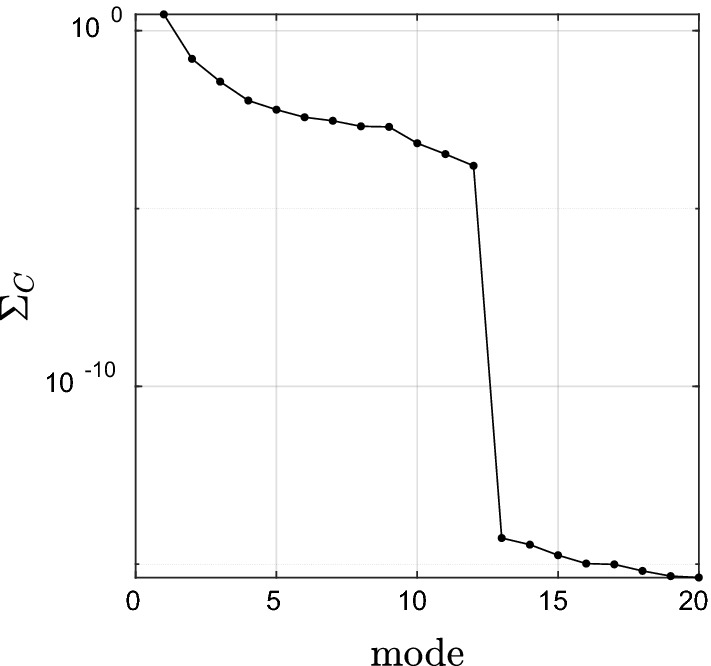
Canonical correlations between CO_2_ concentration and EWH anomaly from GRACE.

**Figure 7:**
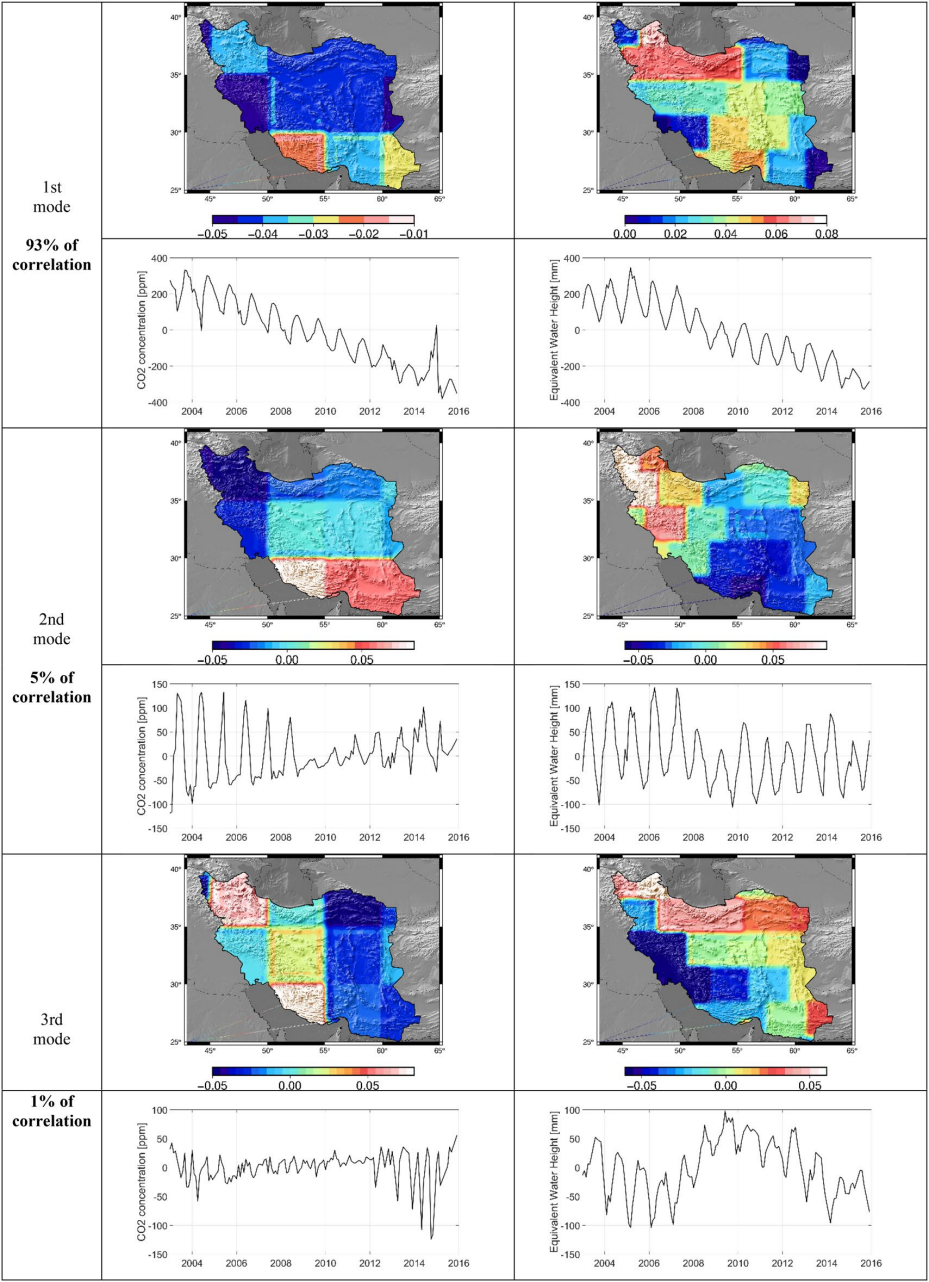
Results of CCA analysis between CO_2_ and EWH anomaly for the first three modes carrying 99% of the correlation. For each mode, time series and maps of canonical modes are shown for both CO_2_ concentration (left) and EWH anomaly from GRACE (right).

**Figure 8:**
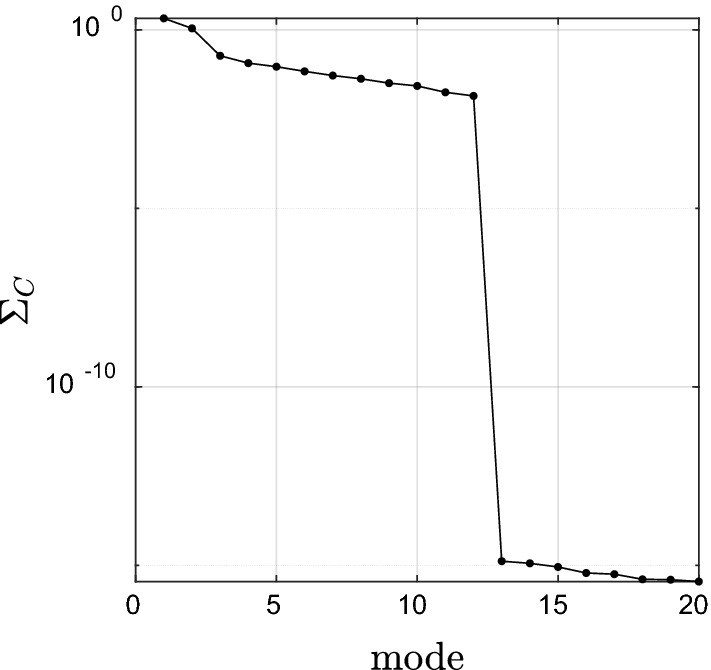
Canonical correlation between the time series of CO_2_ and precipitation

The canonical correlations were determined between two variables series of CO_2_ and EWH anomaly (X independent) and (Y dependent), respectively. Figures 6 and 7 shows the singular values of the cross-covariance between CO_2_ concentration and EWH anomaly highlighting that 99% percent of the correlation lies within the first three modes. Mode 1 with 93% of the canonical correlation between water storage anomaly and CO_2_ concentration captures a combination of trend and seasonal variations, visible in the projection of the obtained canonical mode V_X_, V_Y_ (Fig. 7). The obtained U_X_, U_Y_ represent the spatial pattern of the captured correlation. It is interesting to observe that the water storage changes in the northern part of Iran, except Khorasan and West Azerbaijan Provinces, receive a stronger influence from the increase in CO_2_ concentration. A reason for such a strong pattern in this region might be due to the effect of industrial regions and high population density in Tehran and Alborz province with a high rate of human activities and CO_2_ footprint. On the other, Hyrcanian forest in northern Iran as the main sink of CO_2_ can have a significant role in forming such a relationship. At the same time, one can observe that the CO_2_ concentration and EWH anomaly are less coupled in southern part of Iran along the coast of the Persian Gulf and the Sea of Oman. One reason might be the gas flaring along the coast of the Persian Gulf, especially in Bushehr province that has a strong contribution to greenhouse gas emission^55^. Humphery et al.^35^ showed that TWS based on GRACE data had good correlation with CO_2_ growth rate in semi-arid regions. Mode 2 with 5% of the correlation shows an annual variation without any significant long-term behavior with a stronger dependency in North-West Iran in Azerbaijan province. It is important to mention that on average, CO_2_ values are larger at mid to high northern latitudes, which could be related to various carbon sources and sinks and global winds patterns. In fact, winds in this region are not fast and strong enough to mix all the industrial/anthropogenic CO_2_ emissions from the northern latitude to the south latitude^56^. Moreover, the high dependency between CO_2_ and EWH anomaly in the west of Iran might be due to the significant agricultural activities in this area, which leads to the groundwater depletion with a consequence of an increase in the rate of CO_2_ emission^57^. Humphrey et al.^35^ found that the relationship between temperature and CO_2_ growth rate was not significant in global scale. They also observed that CO_2_ growth rate varied by temperature and total water storage but the role of TWS is more significant than temperature. Zhou et al.^58^ investigated the role of important factors on the potential evapotranspiration variation over China. Their results showed radiation, temperature and CO_2_ concentration were significant factors on potential evapotranspiration, respectively (Fig. 8).

#### CCA between CO_2_ and precipitation

**Figure 9:**
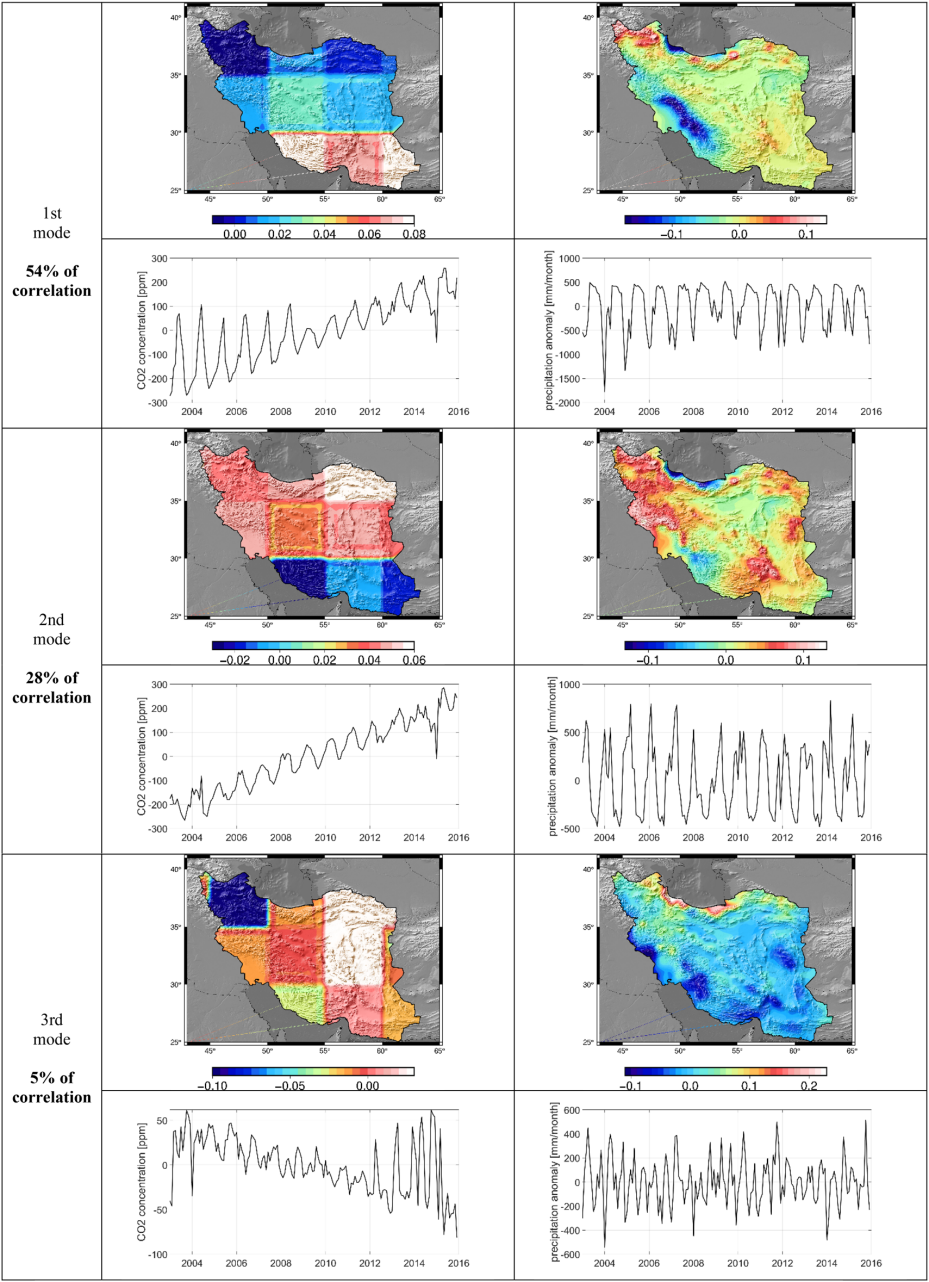
Results of CCA analysis between CO_2_ and Precipitation for the first three modes carrying 87% of the correlation. For each mode, time series and maps of canonical modes are shown for both CO_2_ concentration (left) and precipitation (right).

The canonical correlation between precipitation and CO_2_ datasets does not reveal a strong canonical mode like the one between CO_2_ and EWH anomaly. In this case, mode 1 carries 54% of the correlation between CO_2_ and precipitation with a clear seasonal behavior (Fig. 8 and 9). While both temporal variations of the first mode, show a clear coupled seasonal variation, the temporal behavior of precipitation data does not show any long-term behavior. The spatial patterns of the first mode allow us to identify an interesting finding, where the precipitation in the western part of Zagros Mountains and Guilan province seems to be positively correlated with CO_2_. Moreover, Hyrcanian forest and Zagrous forest in northern and western Iran has a significant role in carbon absorption as the natural sink in warm seasons. Decreasing CO_2_ in the growing season by the photosynthesis process in western and northern Iran is simultaneous with lower precipitation in this area. Meanwhile, severe amplitude CO_2_ and precipitation were observed from 2003 to 2006. The mode 2 with a 28% of correlation also represents a seasonal behavior with a totally different spatial pattern. It reflects a negative strong correlation between seasonal variations of precipitation over high mountains coupled with the CO_2_ concentration. The northern and western regions of Iran have highlands such as Alborz and Zagrous Mountains, respectively, which cause low temperatures and the formation of wind currents in these regions. Air currents have an important role in order to transfer high amounts of CO_2_ to the east and southeast of Iran^33^. Mousavi et al.^59^ found the negative correlation between XCO_2_ and precipitation over Iran from 2003 to 2020 in different months. They observed the same correlation between atmospheric CO_2_ and vegetation density. Costa et al.^48^ applied the empirical model for daily XCO_2_ estimation in Brazil. Their results showed that radiation, sun induced chlorophyll fluorescence and relative humidity had the most important rule in modeling. Therefore, it is expected to observe a negative dependency between CO_2_ concentration and precipitation in this area. Moreover, high precipitation over highland in Iran seems to be negatively correlated with the CO_2_ concentration along the coasts of the Persian Gulf and Sea of Oman. The mode 3 with only 5% canonical correlation reveals a semi-annular dependency with a negative correlation between CO_2_ concentration and precipitation, where stronger amplitudes are seen along the coast of Caspian Sea. In general, the results seem to show that precipitation in the highlands and on the peaks is not more correlated to the long- and short-term variations in CO_2_ concentration. However, we do not observe a rapid decrease or increase in precipitation due to CO_2_ concentration, as expressed in some previous studies (Andrews et al.^60^; Ref.^61^. Though atmospheric CO_2_ variation on global climate has a significant effect on surface water and groundwater (water cycling), evapotranspiration and patterns of precipitation and runoff in natural ecosystems, we did not observe a strong correlation between carbon change and precipitation.

#### CCA between CO_2_ and evapotranspiration

**Figure 10:**
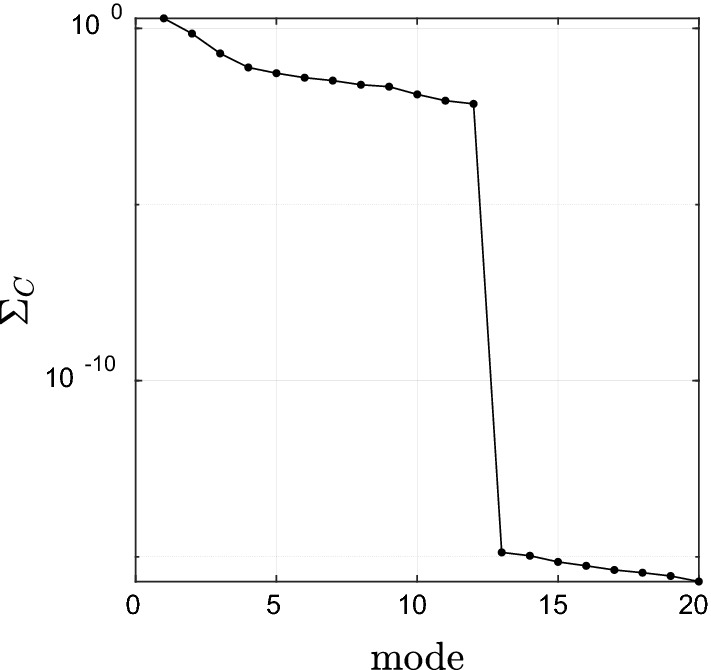
Canonical correlation between the time series of CO_2_ and evapotranspiration.

**Figure 11:**
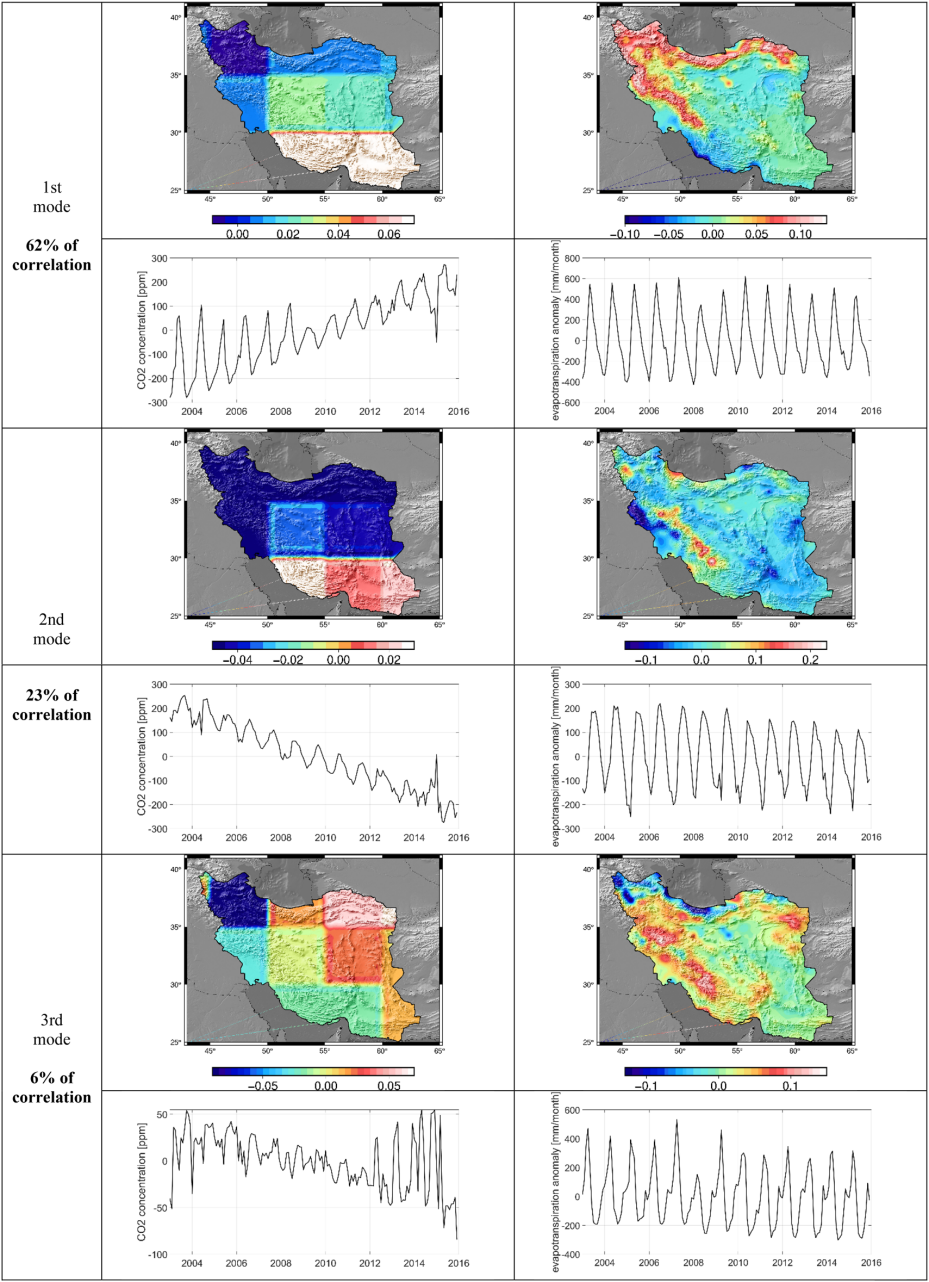
Results of CCA analysis between CO_2_ and evapotranspiration for the first three modes carrying 91% of the correlation. For each mode, time series and maps of canonical modes are shown for both CO_2_ concentration (left) and evapotranspiration (right).

The results of canonical correlation between time series of CO_2_ and evapotranspiration, (X independent and Y dependent) respectively are shown in Figs. 10 and 11. The first three modes carry 91% of the correlation between these two datasets. The first mode extracts an interesting pattern of evapotranspiration over the mountainous regions of Iran along with the Zagros and Alborz mountains in the west and north of Iran. Increasing evapotranspiration with latitude was observed in the snow-free mountains in the hot season. Some meteorological parameters such as cloudiness, surface air pressure, wind speed, solar radiation, and temperature have important effects on evapotranspiration and may lead to an increase in evapotranspiration with latitude^62^. On the other hand, CO_2_ gas has a high density and is heavier than the air. Therefore, when it is released into the atmosphere it will lead to low elevation. This issue is observed in the first mode and represents a similar pattern to the first mode of precipitation. Mode 2 shows two distinct spatial patterns for CO_2_, where the CO_2_ concentration shows a negative trend above the latitude 30° and a positive trend below the latitude 30°. It is related to the variation of tropospheric CO_2_ concentration with geographic latitude. The mode 2 with such a pattern is canonically correlated with evapotranspiration in the high mountains of Zagrous and Lake Urmia. Different microalga species play significant role in carbon sequestration in saline lakes. The presence of these microalgae in Iran’s salt lakes such as Lake Urmia, Qum lake, and Maharlue lake and also shallow-marine limestone in Zagrous Mountain have an effective impact on the carbon cycle^63^. Although CCA does not show any significant long-term behavior in evapotranspiration data correlated with the positive trend in CO_2_, only a slightly positive trend is visible in the third mode (the spatial map shows negative numbers turning the negative trend in the time series in positive trend). A possible reason for such a slight positive trend is the change in temperature due to climate change and change in net radiation (Oguntunde et al.^64^). This result is in line with a previous study by Tabari and Marofi^65^, who showed a weak inverse relationship between potential evaporation and precipitation. This weak positive trend, however, shows a special spatial pattern mainly distributed over the agricultural areas including Lake Urmia basin and Guilan, Mazandaran, and Golestan provinces (see the map of mode 3). Also, some researches showed that the increasing atmospheric CO_2_ concentration has direct and indirect effects vegetation cover and hydrology. The serious evapotranspiration reduction under increasing CO_2_ concentration was observed due to its role in reducing crop water requirements^66^. Liao et al.^67^ used the general circulation models under different RCP scenarios in order to project the evapotranspiration. They showed that evapotranspiration will decrease under doubled CO_2_ concentration in China cropland. Pan et al. showed that high temperature and high precipitation caused to increase in the evapotranspiration until 2100. They also indicated that atmospheric CO_2_ concentration is accounting for decreasing evapotranspiration in future specially in central and western Asia.

### Regression modeling between total water storage and CO_2_/water discharge/water consumption

In stepwise regression, the first variable is entered into the model is based on the maximum effect, and if other variables can significantly affect the dependent variable, they will enter into the model in the next steps. The dependent variable (Y) in this study is the total water storage and the independent variables (X) are carbon dioxide, the statistical information related to deep, semi-deep well discharges, springs, aqueducts, and the water consumption in agricultural, domestic, and industrial sectors. The adjusted coefficient of determination (Adjusted R^2^) shows that CO_2_ has a higher degree of confidence than the coefficient of determination (R^2^), which is estimated to be 0.9 in the present study.

According to Table 2, the carbon dioxide variable has the greatest effect on the output of the regression model. The results showed that the increase or decrease in atmospheric CO_2_ as an effective factor in the water resources can be calibrated in terms of precipitation, evaporation, and transpiration. In the other words, carbon dioxide as an important greenhouse gas in global warming with a direct or an indirect effect on precipitation, temperature, evaporation, and transpiration can be considered as an influential climatic variable for research of Iran’s water resources Many studies focused on the effect of climate change on hydrology parameters in present and future such as Saifullah et al.^24^, Zhuang et al.^68^, Umair et al.^69^, and Ougahi^70^.

$${\text{Y}} = - {6}.{\text{51X}} + {2486}.0{6}$$

Y is equivalent water height, X is CO_2_ concentration.

**Table 2:** Regression modeling between the total water storage and carbon dioxide changes.

R	R square	Adjusted R square	F	Sig	Beta	
					CO_2_	Constant
0.956	0.914	0.907	117.613	0.000	− 0.956	2486.06

## Conclusions

We observed an increasing trend associated with sinusoidal fluctuations in atmospheric carbon dioxide concentration from 2003 to 2015. The highest CO_2_ concentration occurred in spring and the lowest value was observed in summer**.** These results were consistent with Keeling curve data. In terms of spatial distribution, the highest carbon dioxide concentration was observed in southern and southeastern Iran and then in the southwest and center of Iran. Time series analysis showed that the lowest monotonic trend occurs in the pixels that cover the north and northwest of the country. A similar analysis of EWH anomaly showed a decreasing trend over the whole of Iran. During this time period, sinusoidal and decreasing oscillations represented changes in EWH anomaly.

Further, we investigated the dependencies between atmospheric CO_2_ (X independent variable) and EWH anomaly, precipitation, and evapotranspiration (Y dependent variable) by CCA. The results show that the water storage changes in the northern part of Iran, except Khorasan and West Azerbaijan Provinces, receive a stronger influence from the increase in CO_2_ concentration. Such result can be attributed to metropolitan and industrial cities such as Tehran and Alborz provinces with the high rate of carbon emission in day and night time. Our analysis show that the canonical correlation between precipitation and CO_2_ datasets does not highlight a strong canonical mode like the one between CO_2_ and EWH anomaly. According to the first three modes, the correlation between CO_2_ and precipitation was considered in highland areas. Unlike Zagrous Mountain and Guilan province, a negative correlation was observed between CO_2_ and precipitation along the southern coasts in Iran. Our results show that evapotranspiration over the mountainous regions of Iran along with the Zagrous and Alborz mountains in the west and north of Iran is highly correlated with the CO_2_ concentration in the north-western part of Iran. Plants regulated the gaseous exchange with ambient air by their leave’s stomata. CO_2_ uptake and water vapor released by vegetation in the photosynthetic process are dependent on air temperature. Whereas air temperature changes gradually with altitude and geographical latitude, we observe the spatial pattern in CO_2_ and evapotranspiration dependency over Iran. It is clear that the variety of soil types, soil moisture, vegetation type, and vegetation density in the different parts of Iran had significant roles in the formation of these results. Our CCA results provide a new perspective on the application of remotely sensed TWS for regional carbon cycle analysis.

Whereas, carbon dioxide as an important greenhouse gas in global warming has a direct or indirect impact on precipitation, temperature, evapotranspiration, and transpiration, affecting changes in EWH anomaly by reducing depletion of water resources, can be used for reducing the carbon dioxide (CO_2_) emissions. Our results demonstrate that total water storage is linked to changes in atmospheric CO_2_ concentration, evapotranspiration and precipitation on the regional scale. The importance of CO_2_ concentration is more effective than can be distinguished from evapotranspiration and precipitation on TWS. Moreover, conserving natural ecosystems such as Hyrcanian forest and Zagrous forest as carbon sink together with sustainable management of cropland especially in western and northern Iran will have an effective and long-term impact of CO_2_ variation. On the other hand, there seem to be a bilateral relationship between CO_2_ and total water storage. So change in the atmospheric CO_2_ can be effected on water storage and the depletion of groundwater may cause CO_2_ emission. The environmental challenges of groundwater depletion in Iran is harsh as well as the associated CO_2_ emissions, and hence, there is a necessity for a rule of groundwater extraction. Whereof the groundwater extraction is been converted to serious challenge in Iran, the mitigation policy in groundwater withdrew is crucial to Iran’s environmental policies and carbon management.

However, because the CO_2_ data are very coarsely resolved and the focus of this study was on determining the link of climatic parameters and EWH with CO_2_ data, we do not believe that alternating between different data sets would change the results drastically. However, it is important to note that these correlations do not necessarily mean a direct influence. Also, it should be noted that these relationships are tangible at the macro level, while management practices are more influential in changing water resources, especially groundwater at the local scale. Meanwhile the atmospheric CO_2_ concentration is strongly influenced by industrial production as well as human activities (land use change, fossil fuel combustion …) and the same is true for terrestrial water storage (water transfer project, land use change, dam construction …). This research highlighted a novel perspective in which variations in the interplay between atmospheric CO_2_ and TWS based on the potential effect of climate-driven oscillation in hydrology by remotely sensed data for large-scale carbon cycle research. In future work, it is recommended to evaluate the anthropogenic factors for better interpretation and the relationship among atmospheric CO_2_ concentration and EWH anomaly, precipitation and evaporation for different seasons over Iran.

## Data Availability

The datasets analysed during the current study are not publicly available due their containing information that could compromise the privacy of research participants (water consumption and groundwater extraction data) but are available from the corresponding author on reasonable request.
